# Benzene-1,2-diaminium bis­(4-methyl­benzene-1-sulfonate)

**DOI:** 10.1107/S2414314620001005

**Published:** 2020-01-31

**Authors:** Kedar U. Narvekar, Bikshandarkoil R. Srinivasan

**Affiliations:** a School of Chemical Sciences, Goa University PO, Goa 403206, India; Howard University, USA

**Keywords:** crystal structure, benzene-1,2-diaminium dication, 4-methyl­benzene-1-sulfonate anion, organic salt

## Abstract

The structure of the title salt, C_6_H_10_N_2_^2+^·2C_7_H_7_O_3_S^−^, consists of a unique benzene-1,2-diaminium dication charge balanced by a pair of crystallographically independent 4-methyl­benzene-1-sulfonate anions. The cations and anions are inter­linked by several N—H⋯O hydrogen bonds.

## Abstract 2

In the structure of the title compound, which consists of a unique benzene-1,2-diaminium dication charge-balanced by a pair of crystallographically independent 4-methyl­benzene-1-sulfonate anions, the cations and anions are inter­linked by several N—H⋯O hydrogen bonds.

## Graphical abstract

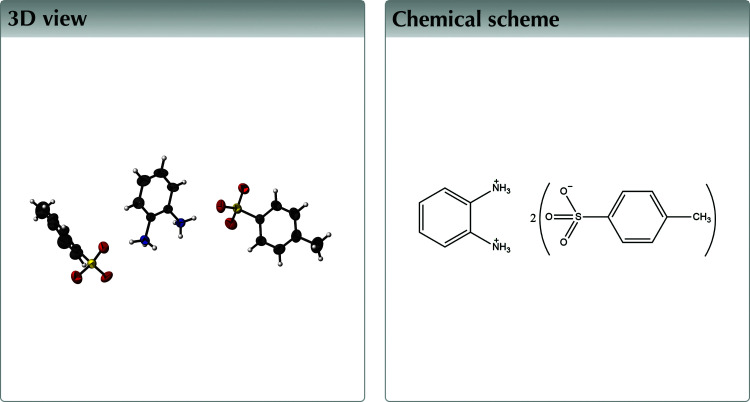

## Structure description

The aromatic di­amine, benzene-1,2-di­amine also known as *o*-phenyl­enedi­amine, can function as a neutral ligand and bind to a metal *via* both amine N atoms (Koizumi & Fukuju, 2011 ▸; Guillén *et al.*, 2018 ▸) or by a single nitro­gen in a monodentate fashion (Nelson *et al.*, 1982 ▸; Dickman, 2000 ▸). In addition, benzene-1,2-di­amine can function as a charge-balancing dication in which both the amine N atoms are protonated (Raghavaiah *et al.*, 2006 ▸; Powers & Geiger, 2019 ▸) or as a monocation (Raghavaiah *et al.*, 2005 ▸; Mishra & Pallepogu, 2018 ▸). The structural diversity of the compounds of benzene-1,2-di­amine in neutral or cationic form is highlighted by the results of a survey of the Cambridge Structural Database (CSD; Groom *et al.*, 2016 ▸), which had more than 220 hits for the above three types of compounds. Of these, a total of 79 deposits do not contain any metal ions and correspond to crystal structures containing only diprotonated benzene-1,2-diaminium cations (47 hits) and monoprotonated 2-amino­anilinium cations (55 hits). An example of a mol­ecular salt of 4-methyl­benzene-1-sulfonic acid containing both mono and diprotonated cations, namely 2-amino­anilinium benzene-1,2-diaminium tris­(4-methyl­benzene-1-sulfonate) (**2**) has been reported recently (Amirthakumar *et al.*, 2018 ▸).

**Figure 1:**
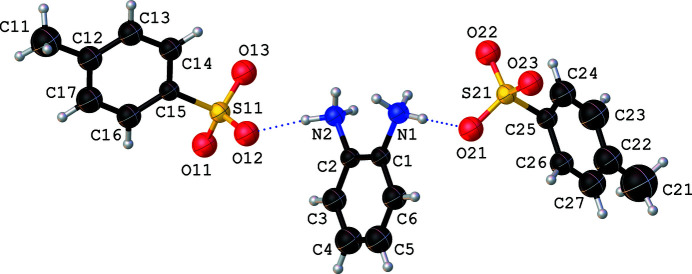
The crystal structure of **1** showing the atom-labelling scheme. Blue dotted lines indicate hydrogen bonds. Displacement ellipsoids are drawn at the 50% probability level. H atoms are shown as spheres of arbitrary radii.

In this report, we describe the crystal structure of the title compound, which was obtained by an aqueous reaction of the aromatic di­amine with 4-methyl­benzene-1-sulfonic acid in a 1:2 molar ratio, unlike **2**, which was isolated from a 1:1 reaction. The asymmetric unit of the title compound consists of an unique benzene-1,2-diaminium dication charge-balanced by a pair of crystallographically independent 4-methyl­benzene-1-sulfonate anions (Fig. 1 ▸) with all atoms located on general positions. The geometric parameters of the unique dication and the crystallographically independent anions are in normal ranges and are in agreement with reported data (Powers & Geiger, 2019 ▸).

**Figure 2:**
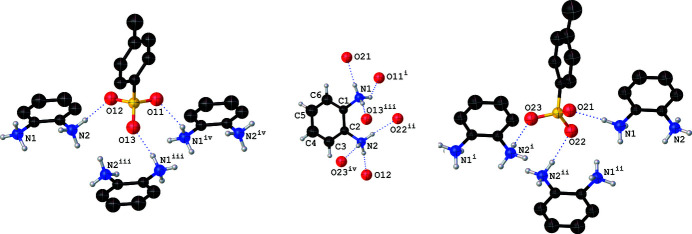
The hydrogen-bonding scheme around the two unique anions (left and right). For clarity, the H atoms of the aromatic ring are not shown. The hydrogen-bonding environment of the dication (middle) shows only the acceptor oxygen atoms of the unique anions. Symmetry codes: (i) *x*, *y* + 1, *z*; (ii) −*x* + 1, −*y* + 1, −*z* + 1; (iii) −*x* + 1, −*y*, −*z* + 1; (iv) *x*, *y* − 1, *z*.

**Figure 3:**
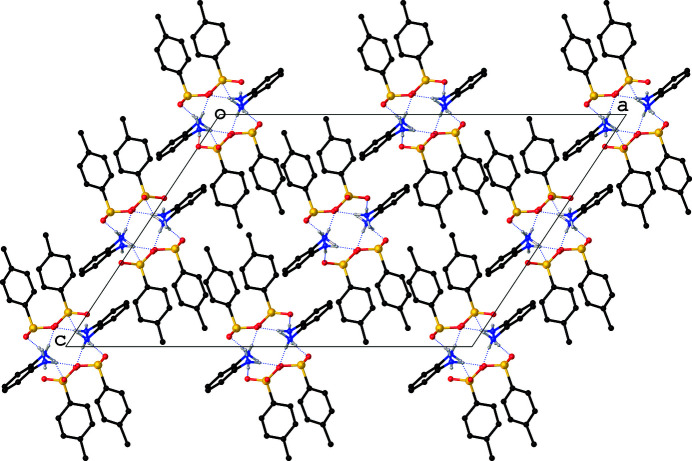
A view along *b* axis of the unit-cell packing showing the inter­linking of the dications with the monocations *via* N—H⋯O hydrogen bonds which are shown as dashed lines. For clarity, the H atoms attached to the C atoms are not shown.

All six oxygen atoms attached to the sulfur atom of the sulfonate moiety of the anion function as hydrogen-bond acceptors while the H atoms attached to the N atoms of the dication function as hydrogen-bond donors, resulting in a total of eight N—H⋯O hydrogen bonds of which six are inter­molecular (Table 1 ▸). It is inter­esting to note that the dications and the unique anions are inter­linked only *via* N—H⋯O hydrogen bonds, unlike in **2** for which both N—H⋯O and C—H⋯O hydrogen bonds were reported. Each anion is linked to three symmetry-related dications (Fig. 2 ▸) while each dication is hydrogen-bonded to six symmetry-related anions. The net result of the hydrogen-bonding inter­actions is the inter­linking of the cations with the anions, resulting in alternating layers of cations and anions parallel to [010] (Fig. 3 ▸).

## Synthesis and crystallization

Freshly recrystallized benzene-1,2-di­amine (108 mg, 1 mmol) was dissolved in double-distilled water (10–15 ml) by heating the mixture. Into this, an aqueous solution of 4-methyl­benzene-1-sulfonic acid (380 mg, 2 mmol) was added. The reaction mixture was heated to boiling and a pinch of activated charcoal was added. The hot solution was filtered and the clear filtrate was left aside for crystallization. After a few days, crystals of the title compound **1** slowly separated. The crystals were filtered and air dried. Yield 50%.

## Refinement

Crystal data, data collection and structure refinement details are summarized in Table 2 ▸.

## Supplementary Material

Crystal structure: contains datablock(s) I, global. DOI: 10.1107/S2414314620001005/bv4029sup1.cif

Structure factors: contains datablock(s) I. DOI: 10.1107/S2414314620001005/bv4029Isup3.hkl

Supporting information file. DOI: 10.1107/S2414314620001005/bv4029Isup3.cml

CCDC reference: 1979912

Additional supporting information:  crystallographic information; 3D view; checkCIF report

## Figures and Tables

**Table 1:** Hydrogen-bond geometry (Å, °)

*D*—H⋯*A*	*D*—H	H⋯*A*	*D*⋯*A*	*D*—H⋯*A*
N1—H1*A*⋯O21	0.89	1.92	2.8062 (19)	175
N1—H1*B*⋯O11^i^	0.89	2.07	2.7509 (19)	133
N1—H1*B*⋯O22^ii^	0.89	2.31	2.9017 (18)	124
N1—H1*C*⋯O13^iii^	0.89	1.89	2.7733 (19)	170
N2—H2*A*⋯O13^iii^	0.89	2.46	2.9128 (19)	112
N2—H2*A*⋯O23^iv^	0.89	1.97	2.7820 (18)	151
N2—H2*B*⋯O22^ii^	0.89	1.98	2.8611 (19)	173
N2—H2*C*⋯O12	0.89	1.84	2.7224 (19)	175

Symmetry codes: (i) 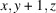 ; (ii) 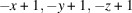 ; (iii) 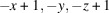 ; (iv) 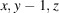 .

**Table 2:** Experimental details

Crystal data	
Chemical formula	C_6_H_10_N_2_^2+^·2C_7_H_7_O_3_S^−^
*M*_r_	452.53
Crystal system, space group	Monoclinic, *C*2/*c*
Temperature (K)	293
*a*, *b*, *c* (Å)	29.1537 (9), 8.8739 (3), 19.9919 (6)
β (°)	123.621 (1)
*V* (Å^3^)	4306.9 (2)
*Z*	8
Radiation type	Mo *K*α
μ (mm^−1^)	0.29
Crystal size (mm)	0.56 × 0.15 × 0.12

Data collection	
Diffractometer	Bruker D8 Quest ECO
Absorption correction	Multi-scan (*SADABS*; Bruker, 2018 ▸)
*T*_min_, *T*_max_	0.696, 0.746
No. of measured, independent and observed [*I* > 2σ(*I*)] reflections	59056, 6568, 4705
*R*_int_	0.046
(sin θ/λ)_max_ (Å^−1^)	0.715

Refinement	
*R*[*F* ^2^ > 2σ(*F* ^2^)], *wR*(*F* ^2^), *S*	0.040, 0.126, 1.07
No. of reflections	6568
No. of parameters	276
H-atom treatment	H-atom parameters constrained
Δρ_max_, Δρ_min_ (e Å^−3^)	0.34, −0.39

Computer programs: *APEX3* and *SAINT* (Bruker, 2018 ▸), *SHELXT2014* (Sheldrick, 2015*a* ▸), *SHELXL2018* (Sheldrick, 2015*b* ▸), *OLEX2* (Dolomanov *et al.*, 2009 ▸) and *shelXle* (Hübschle *et al.*, 2011 ▸).

## full crystallographic data

### Crystal data

**Table:**

C_6_H_10_N_2_^2+^·2C_7_H_7_O_3_S^−^	*F*(000) = 1904
*M**_r_* = 452.53	*D*_x_ = 1.396 Mg m^−^^3^
Monoclinic, *C*2/*c*	Mo *K*α radiation, λ = 0.71073 Å
*a* = 29.1537 (9) Å	Cell parameters from 9968 reflections
*b* = 8.8739 (3) Å	θ = 2.9–29.9°
*c* = 19.9919 (6) Å	µ = 0.29 mm^−^^1^
β = 123.621 (1)°	*T* = 293 K
*V* = 4306.9 (2) Å^3^	Block, colourless
*Z* = 8	0.56 × 0.14 × 0.12 mm

### Data collection

**Table:**

Bruker D8 Quest ECO diffractometer	4705 reflections with *I* > 2σ(*I*)
Radiation source: Sealed Tube	*R*_int_ = 0.046
φ and ω scans	θ_max_ = 30.5°, θ_min_ = 2.9°
Absorption correction: multi-scan (SADABS; Bruker, 2018)	*h* = −41→41
*T*_min_ = 0.696, *T*_max_ = 0.746	*k* = −12→12
59056 measured reflections	*l* = −28→28
6568 independent reflections	

### Refinement

**Table:**

Refinement on *F*^2^	Hydrogen site location: inferred from neighbouring sites
Least-squares matrix: full	H-atom parameters constrained
*R*[*F*^2^ > 2σ(*F*^2^)] = 0.040	*w* = 1/[σ^2^(*F*_o_^2^) + (0.0525*P*)^2^ + 2.9966*P*] where *P* = (*F*_o_^2^ + 2*F*_c_^2^)/3
*wR*(*F*^2^) = 0.126	(Δ/σ)_max_ = 0.001
*S* = 1.07	Δρ_max_ = 0.34 e Å^−^^3^
6568 reflections	Δρ_min_ = −0.39 e Å^−^^3^
276 parameters	Extinction correction: SHELXL2018 (Sheldrick, 2015b), Fc^*^=kFc[1+0.001xFc^2^λ^3^/sin(2θ)]^-1/4^
0 restraints	Extinction coefficient: 0.0026 (3)

### Special details

**Table:**

Geometry. All esds (except the esd in the dihedral angle between two l.s. planes) are estimated using the full covariance matrix. The cell esds are taken into account individually in the estimation of esds in distances, angles and torsion angles; correlations between esds in cell parameters are only used when they are defined by crystal symmetry. An approximate (isotropic) treatment of cell esds is used for estimating esds involving l.s. planes.
Refinement. All hydrogen atoms were located in appropriate positions and were included in calculated positions and refined with a riding model for both C—H and N—H protons. C–H distances ranged from = 0.93 and 0.96 Å for aromatic and methyl H atoms, respectively, and 0.89 for NH_3_^+^ H atoms with *U*_iso_(H) = 1.2 *U*_eq_(C-aromatic) and *U*_iso_(H) = 1.5 *U*_eq_(*C*-methyl, NH_3_^+^).

### Fractional atomic coordinates and isotropic or equivalent isotropic displacement parameters (Å^2^)

**Table:**

	*x*	*y*	*z*	*U*_iso_*/*U*_eq_	
N1	0.52597 (6)	0.42385 (15)	0.43447 (8)	0.0399 (3)	
H1A	0.516000	0.511465	0.408451	0.060*	
H1B	0.552390	0.439398	0.485886	0.060*	
H1C	0.497014	0.381592	0.430938	0.060*	
N2	0.54019 (6)	0.10069 (15)	0.46813 (8)	0.0374 (3)	
H2A	0.505767	0.066255	0.439229	0.056*	
H2B	0.543473	0.168126	0.503495	0.056*	
H2C	0.563230	0.024462	0.494094	0.056*	
C1	0.54643 (6)	0.32416 (17)	0.39869 (9)	0.0349 (3)	
C2	0.55343 (6)	0.17140 (17)	0.41510 (9)	0.0344 (3)	
C3	0.57224 (8)	0.0796 (2)	0.37927 (12)	0.0505 (4)	
H3	0.576521	−0.023317	0.389900	0.061*	
C4	0.58462 (11)	0.1411 (3)	0.32770 (14)	0.0661 (6)	
H4	0.597256	0.079592	0.303508	0.079*	
C5	0.57830 (11)	0.2935 (3)	0.31201 (15)	0.0664 (6)	
H5	0.586983	0.334936	0.277609	0.080*	
C6	0.55909 (9)	0.3848 (2)	0.34726 (12)	0.0529 (4)	
H6	0.554660	0.487571	0.336310	0.064*	
S11	0.60971 (2)	−0.26457 (4)	0.57918 (2)	0.03904 (11)	
O11	0.61408 (5)	−0.40103 (15)	0.54362 (8)	0.0562 (3)	
O12	0.61358 (6)	−0.12891 (15)	0.54200 (8)	0.0563 (3)	
O13	0.56134 (5)	−0.26132 (16)	0.58310 (9)	0.0573 (4)	
C11	0.81185 (11)	−0.2503 (3)	0.92035 (15)	0.0827 (8)	
H11A	0.833550	−0.338591	0.929269	0.124*	
H11B	0.801302	−0.248348	0.958103	0.124*	
H11C	0.833111	−0.161970	0.927333	0.124*	
C12	0.76068 (8)	−0.2532 (2)	0.83564 (12)	0.0540 (5)	
C13	0.71405 (8)	−0.1716 (2)	0.81418 (11)	0.0541 (5)	
H13	0.713844	−0.113580	0.852757	0.065*	
C14	0.66758 (7)	−0.1744 (2)	0.73639 (11)	0.0460 (4)	
H14	0.636634	−0.118206	0.722766	0.055*	
C15	0.66778 (6)	−0.26174 (17)	0.67934 (10)	0.0363 (3)	
C16	0.71387 (7)	−0.3444 (2)	0.69966 (12)	0.0523 (4)	
H16	0.713945	−0.403424	0.661270	0.063*	
C17	0.76014 (8)	−0.3390 (3)	0.77780 (13)	0.0616 (5)	
H17	0.791258	−0.394165	0.791294	0.074*	
S21	0.45059 (2)	0.76496 (4)	0.35286 (2)	0.03714 (11)	
O21	0.49995 (5)	0.70837 (16)	0.36096 (8)	0.0547 (3)	
O22	0.44084 (6)	0.69248 (16)	0.40921 (7)	0.0559 (3)	
O23	0.44971 (6)	0.92776 (14)	0.35764 (8)	0.0565 (4)	
C21	0.26881 (15)	0.5602 (4)	0.01997 (17)	0.1233 (14)	
H21A	0.233080	0.582893	0.009049	0.185*	
H21B	0.272805	0.452969	0.018852	0.185*	
H21C	0.272661	0.606499	−0.020056	0.185*	
C22	0.31300 (11)	0.6205 (3)	0.10249 (13)	0.0742 (7)	
C23	0.30782 (11)	0.6004 (3)	0.16652 (16)	0.0813 (7)	
H23	0.275944	0.556751	0.157815	0.098*	
C24	0.34940 (9)	0.6443 (3)	0.24375 (13)	0.0609 (5)	
H24	0.345911	0.627550	0.286660	0.073*	
C25	0.39576 (7)	0.71265 (18)	0.25587 (9)	0.0387 (3)	
C26	0.40091 (9)	0.7360 (2)	0.19192 (11)	0.0496 (4)	
H26	0.432285	0.782254	0.200184	0.060*	
C27	0.35939 (11)	0.6905 (3)	0.11584 (12)	0.0665 (6)	
H27	0.362871	0.707427	0.072940	0.080*	

### Atomic displacement parameters (Å^2^)

**Table:**

	*U*^11^	*U*^22^	*U*^33^	*U*^12^	*U*^13^	*U*^23^
N1	0.0434 (7)	0.0323 (6)	0.0424 (7)	0.0035 (5)	0.0229 (6)	−0.0005 (5)
N2	0.0435 (7)	0.0346 (6)	0.0376 (7)	0.0003 (5)	0.0246 (6)	0.0022 (5)
C1	0.0357 (7)	0.0340 (7)	0.0356 (7)	−0.0015 (6)	0.0202 (6)	−0.0014 (6)
C2	0.0390 (8)	0.0329 (7)	0.0352 (7)	−0.0019 (6)	0.0230 (6)	−0.0009 (6)
C3	0.0707 (12)	0.0390 (9)	0.0597 (11)	0.0038 (8)	0.0473 (10)	−0.0019 (8)
C4	0.0979 (17)	0.0598 (12)	0.0792 (15)	0.0033 (11)	0.0732 (14)	−0.0036 (11)
C5	0.0924 (16)	0.0672 (13)	0.0737 (14)	−0.0001 (12)	0.0673 (14)	0.0091 (11)
C6	0.0669 (12)	0.0435 (9)	0.0623 (11)	−0.0018 (8)	0.0444 (10)	0.0082 (8)
S11	0.03285 (19)	0.0358 (2)	0.0398 (2)	0.00174 (14)	0.01463 (16)	−0.00139 (15)
O11	0.0498 (7)	0.0493 (7)	0.0513 (7)	0.0054 (6)	0.0165 (6)	−0.0134 (6)
O12	0.0558 (8)	0.0497 (7)	0.0522 (7)	0.0056 (6)	0.0227 (6)	0.0147 (6)
O13	0.0324 (6)	0.0628 (9)	0.0694 (9)	−0.0034 (5)	0.0237 (6)	−0.0094 (7)
C11	0.0602 (14)	0.095 (2)	0.0504 (12)	0.0003 (12)	0.0039 (11)	−0.0007 (12)
C12	0.0433 (9)	0.0557 (11)	0.0440 (10)	−0.0025 (8)	0.0122 (8)	0.0027 (8)
C13	0.0534 (11)	0.0615 (12)	0.0427 (9)	−0.0035 (9)	0.0236 (8)	−0.0089 (8)
C14	0.0403 (8)	0.0516 (10)	0.0449 (9)	0.0032 (7)	0.0229 (7)	−0.0038 (7)
C15	0.0320 (7)	0.0356 (7)	0.0390 (8)	−0.0009 (6)	0.0182 (6)	0.0017 (6)
C16	0.0413 (9)	0.0563 (11)	0.0504 (10)	0.0096 (8)	0.0199 (8)	−0.0063 (8)
C17	0.0403 (10)	0.0659 (13)	0.0586 (12)	0.0134 (9)	0.0149 (9)	−0.0009 (10)
S21	0.0452 (2)	0.03159 (19)	0.03394 (19)	0.00202 (14)	0.02151 (17)	0.00074 (13)
O21	0.0502 (7)	0.0599 (8)	0.0523 (7)	0.0147 (6)	0.0274 (6)	0.0096 (6)
O22	0.0741 (9)	0.0606 (8)	0.0383 (6)	−0.0077 (7)	0.0345 (7)	−0.0003 (6)
O23	0.0581 (8)	0.0318 (6)	0.0582 (8)	0.0003 (5)	0.0188 (7)	−0.0062 (5)
C21	0.131 (3)	0.089 (2)	0.0642 (16)	−0.005 (2)	0.0007 (17)	−0.0314 (16)
C22	0.0841 (16)	0.0541 (12)	0.0475 (11)	0.0005 (11)	0.0132 (11)	−0.0131 (9)
C23	0.0700 (15)	0.0772 (17)	0.0764 (16)	−0.0281 (13)	0.0279 (13)	−0.0191 (13)
C24	0.0641 (12)	0.0657 (13)	0.0564 (11)	−0.0176 (10)	0.0355 (10)	−0.0091 (10)
C25	0.0486 (9)	0.0326 (7)	0.0356 (7)	0.0005 (6)	0.0237 (7)	−0.0019 (6)
C26	0.0659 (12)	0.0465 (10)	0.0406 (9)	0.0041 (8)	0.0320 (9)	0.0042 (7)
C27	0.0919 (17)	0.0610 (12)	0.0383 (9)	0.0128 (12)	0.0309 (11)	0.0004 (9)

### Geometric parameters (Å, º)

**Table:**

N1—C1	1.4570 (19)	C13—C14	1.386 (3)
N2—C2	1.4561 (18)	C14—C15	1.382 (2)
C1—C6	1.380 (2)	C15—C16	1.380 (2)
C1—C2	1.383 (2)	C16—C17	1.389 (3)
C2—C3	1.383 (2)	S21—O21	1.4462 (14)
C3—C4	1.380 (3)	S21—O23	1.4490 (13)
C4—C5	1.378 (3)	S21—O22	1.4554 (13)
C5—C6	1.380 (3)	S21—C25	1.7602 (17)
S11—O11	1.4451 (13)	C21—C22	1.521 (3)
S11—O12	1.4517 (14)	C22—C27	1.373 (4)
S11—O13	1.4553 (14)	C22—C23	1.381 (4)
S11—C15	1.7671 (16)	C23—C24	1.390 (3)
C11—C12	1.515 (3)	C24—C25	1.375 (3)
C12—C17	1.377 (3)	C25—C26	1.383 (2)
C12—C13	1.382 (3)	C26—C27	1.379 (3)
			
C6—C1—C2	119.60 (15)	C16—C15—C14	120.25 (16)
C6—C1—N1	118.71 (15)	C16—C15—S11	119.46 (13)
C2—C1—N1	121.69 (13)	C14—C15—S11	120.27 (12)
C3—C2—C1	120.23 (14)	C15—C16—C17	119.57 (18)
C3—C2—N2	117.66 (14)	C12—C17—C16	121.09 (18)
C1—C2—N2	122.10 (13)	O21—S21—O23	113.06 (9)
C4—C3—C2	119.78 (17)	O21—S21—O22	111.63 (9)
C5—C4—C3	120.09 (18)	O23—S21—O22	111.81 (9)
C4—C5—C6	120.07 (18)	O21—S21—C25	105.80 (8)
C5—C6—C1	120.23 (18)	O23—S21—C25	107.28 (8)
O11—S11—O12	112.95 (9)	O22—S21—C25	106.77 (8)
O11—S11—O13	113.27 (8)	C27—C22—C23	118.7 (2)
O12—S11—O13	111.32 (8)	C27—C22—C21	121.4 (3)
O11—S11—C15	106.21 (7)	C23—C22—C21	119.9 (3)
O12—S11—C15	105.73 (8)	C22—C23—C24	121.2 (2)
O13—S11—C15	106.72 (8)	C25—C24—C23	119.1 (2)
C17—C12—C13	118.48 (17)	C24—C25—C26	120.16 (17)
C17—C12—C11	119.6 (2)	C24—C25—S21	121.00 (14)
C13—C12—C11	121.9 (2)	C26—C25—S21	118.79 (14)
C12—C13—C14	121.40 (18)	C27—C26—C25	119.8 (2)
C15—C14—C13	119.22 (17)	C22—C27—C26	121.0 (2)
			
C6—C1—C2—C3	−0.9 (2)	C14—C15—C16—C17	−0.2 (3)
N1—C1—C2—C3	179.13 (16)	S11—C15—C16—C17	178.16 (16)
C6—C1—C2—N2	−179.29 (16)	C13—C12—C17—C16	−0.2 (3)
N1—C1—C2—N2	0.8 (2)	C11—C12—C17—C16	179.8 (2)
C1—C2—C3—C4	0.7 (3)	C15—C16—C17—C12	0.5 (3)
N2—C2—C3—C4	179.15 (19)	C27—C22—C23—C24	−2.5 (4)
C2—C3—C4—C5	0.0 (4)	C21—C22—C23—C24	175.2 (3)
C3—C4—C5—C6	−0.6 (4)	C22—C23—C24—C25	1.8 (4)
C4—C5—C6—C1	0.4 (4)	C23—C24—C25—C26	−0.6 (3)
C2—C1—C6—C5	0.4 (3)	C23—C24—C25—S21	−177.95 (19)
N1—C1—C6—C5	−179.68 (19)	O21—S21—C25—C24	131.47 (17)
C17—C12—C13—C14	−0.3 (3)	O23—S21—C25—C24	−107.57 (17)
C11—C12—C13—C14	179.6 (2)	O22—S21—C25—C24	12.44 (18)
C12—C13—C14—C15	0.6 (3)	O21—S21—C25—C26	−45.95 (16)
C13—C14—C15—C16	−0.3 (3)	O23—S21—C25—C26	75.00 (16)
C13—C14—C15—S11	−178.67 (15)	O22—S21—C25—C26	−164.99 (14)
O11—S11—C15—C16	22.07 (17)	C24—C25—C26—C27	0.0 (3)
O12—S11—C15—C16	−98.17 (16)	S21—C25—C26—C27	177.46 (15)
O13—S11—C15—C16	143.20 (15)	C23—C22—C27—C26	1.9 (4)
O11—S11—C15—C14	−159.58 (14)	C21—C22—C27—C26	−175.7 (2)
O12—S11—C15—C14	80.18 (15)	C25—C26—C27—C22	−0.7 (3)
O13—S11—C15—C14	−38.45 (16)		

### Hydrogen-bond geometry (Å, º)

**Table:**

*D*—H···*A*	*D*—H	H···*A*	*D*···*A*	*D*—H···*A*
N1—H1*A*···O21	0.89	1.92	2.8062 (19)	175
N1—H1*B*···O11^i^	0.89	2.07	2.7509 (19)	133
N1—H1*B*···O22^ii^	0.89	2.31	2.9017 (18)	124
N1—H1*C*···O13^iii^	0.89	1.89	2.7733 (19)	170
N2—H2*A*···O13^iii^	0.89	2.46	2.9128 (19)	112
N2—H2*A*···O23^iv^	0.89	1.97	2.7820 (18)	151
N2—H2*B*···O22^ii^	0.89	1.98	2.8611 (19)	173
N2—H2*C*···O12	0.89	1.84	2.7224 (19)	175

Symmetry codes: (i) *x*, *y*+1, *z*; (ii) −*x*+1, −*y*+1, −*z*+1; (iii) −*x*+1, −*y*, −*z*+1; (iv) *x*, *y*−1, *z*.
